# The Molecular Landscape of Lung Metastasis in Primary Head and Neck Squamous Cell Carcinomas

**DOI:** 10.7759/cureus.57497

**Published:** 2024-04-03

**Authors:** Logalakshmi Thirumani, Mizpha Helan, Vijayaraghavan S, Umargani Jamal Mohamed, Sugumar Vimal, Inamul Hasan Madar

**Affiliations:** 1 Multiomics and Precision Medicine Laboratory, Center for Global Health Research, Saveetha Medical College & Hospital, Saveetha Institute of Medical and Technical Sciences (SIMATS), Chennai, IND; 2 Biochemistry, Saveetha Medical College & Hospital, Saveetha Institute of Medical and Technical Sciences (SIMATS), Chennai, IND

**Keywords:** cancer pathway, genetic oncology, genetic expression, lung cancer, head and neck squamous cell carcinoma (hnscc)

## Abstract

Background

Lung metastasis in head and neck cancer (HNC) patients is a critical concern, often indicating an advanced disease stage and a poor prognosis. This study explores the molecular complexities of such metastases, identifying specific genes and pathways that may serve as valuable targets for diagnosis and treatment. The findings underscore the potential for significantly improved patient outcomes through targeted therapeutic strategies.

Methodology

In this research, we systematically collected raw gene expression data from head and neck squamous cell carcinoma (HNSCC) and lung squamous cell carcinoma (LSCC). By comparing tumorous and normal gene expression profiles from paired patient samples, we identified differentially expressed genes (DEGs). Network analysis helped visualize protein interactions and pinpoint crucial hub genes. Through validation and comparison across several datasets, we identified common DEGs. Additionally, we employed Kaplan-Meier analysis and log-rank testing to examine the relationship between gene expression patterns and patient survival.

Result

The study identified 145 overlapping DEGs in both HNSCC and LSCC, which are crucial for cancer progression and linked to lung metastasis, offering vital targets for personalized therapy by identifying key genes affecting disease development and patient survival. Pathway analyses linked these to lung metastasis, while protein-protein interaction network construction and hub gene identification highlighted genes crucial for development and patient survival, offering targets for personalized therapy.

Conclusion

Identifying key genes and pathways in lung metastasis from HNC, this study highlights potential targets for enhanced diagnosis and therapy. It underscores the crucial role of molecular insights in driving forward personalized treatment approaches and improving patient outcomes.

## Introduction

Head and neck cancer (HNC) represents a group of biologically similar cancers originating from the squamocolumnar junction of the head and neck region, including the oral cavity, pharynx, larynx, paranasal sinuses, and nasal cavity. These malignancies are predominantly squamous cell carcinomas, developing from premalignant lesions influenced by environmental and lifestyle factors. Tobacco use and alcohol consumption are the most significant risk factors, synergistically increasing the risk of HNC. Infection with high-risk human papillomavirus (HPV), particularly HPV-16, is a recognized etiological factor for oropharyngeal cancers. Other risk factors include exposure to ultraviolet radiation, occupational hazards like asbestos, poor oral hygiene, and genetic predisposition. The clinical presentation of HNC can vary but often includes non-healing ulcers, dysphagia, and persistent pain. Diagnosis is made through histopathological examination following a biopsy of the suspicious lesion. Imaging studies such as CT, MRI, and PET scans are used for staging and treatment planning. Treatment modalities for HNC include surgery, radiation therapy, chemotherapy, and targeted biological agents, with the choice of treatment depending on the cancer stage and location [1]. Despite advances in treatment, the prognosis for HNC remains variable, with a five-year survival rate ranging from 40% to 50% for most head and neck sites [2]. The global burden of HNC is substantial, with epidemiological data revealing significant disparities in incidence and mortality rates across different regions and populations. Globally, HNC represents a significant public health burden, with an estimated 890,000 new cases and 450,000 deaths reported annually [3].

One of the main causes of cancer-related morbidity and death is metastasis, or the spread of the disease from its original site to other organs. The lungs are the most common site for distant metastases from HNC, with approximately 70-85% of all distant metastases occurring in the lungs [4]. Globally, the burden of cancer, including HNC, continues to rise. According to GLOBOCAN 2020, there were an estimated 19.3 million new cancer cases and 10 million cancer deaths in 2020 [5]. Lung cancer remains one of the most common and deadly forms of cancer globally, with over 2.2 million new cases reported in 2020. The mortality rate of metastatic head and neck squamous cell carcinoma (HNSCC), which includes those with lung metastasis, is reported to be around 15-20%. This underscores the importance of early detection and treatment of HNC to prevent the development of metastases [6]. To enhance patient outcomes, a deeper comprehension of the particular molecular pathways behind HNC metastasis is required. Differentially expressed genes (DEGs) in HNC and lung cancer have been extensively profiled in recent years; however, few studies identify DEGs particularly linked to HNC lung metastases.

The treatment of HNC patients with lung metastasis poses significant challenges, primarily due to the advanced stage of the disease and the associated poor prognosis. The primary challenge is choosing the most appropriate treatment approach, which could range from aggressive interventions like surgery to systemic therapies such as chemotherapy and radiotherapy, all while considering the patient’s quality of life and potential survival benefits [7]. The concept of oligometastasis, which refers to a limited number of metastatic lesions, further complicates treatment decisions, as it raises the question of whether aggressive treatment of these lesions could potentially extend survival or merely expose patients to unnecessary toxicity [8]. Personalized treatment plans are essential, considering the patient’s overall health, the characteristics of the lung metastases, and their response to previous treatments. Accurate prognostic assessment is also challenging, as it involves evaluating the tumor’s behavior, the patient’s health status, and the expected response to various treatment options.

DEGs in lung cancer and HNC are crucial for understanding the progression of the disease and for developing diagnostic and therapeutic strategies. DEGs can reveal the molecular alterations that drive metastasis, which is essential for identifying potential biomarkers and therapeutic targets. For instance, a study identified seven anoikis-related long non-coding RNAs that could predict prognosis, immune responses, and therapeutic effects in HNSCC patients [9]. Another study found four DEGs that could predict prognosis and microenvironment immune infiltration in lung cancer, which are also relevant for understanding lung metastasis from HNC [10]. Despite advancements in diagnosis and treatment, HNC continues to pose a substantial public health challenge with a variable prognosis. Lung metastasis from HNC marks a critical juncture, denoting advanced disease and poor outcomes. Our research aims to address the gap in understanding the molecular mechanisms driving lung metastasis in HNC, focusing on DEGs between HNC and lung cancer to uncover potential diagnostic and therapeutic targets. This exploration is crucial for enhancing clinical management and patient survival in the context of a rising global cancer burden. Addressing this gap is of paramount importance, as it holds the potential to significantly enhance the clinical management of lung metastasis in HNC patients, ultimately improving patient outcomes. Comparing gene expression profiles between HNC and lung cancer patients is significant for understanding lung metastasis in HNC. This comparison can distinguish primary lung tumors from metastatic HNC lesions, which is crucial for accurate diagnosis and treatment. It may reveal unique molecular signatures associated with lung metastasis, leading to targeted therapy development. Gene expression profiles can enhance prognostic models, allowing better predictions of outcomes and personalized treatments. Understanding the tumor microenvironment differences between primary lung cancers and metastatic HNC can influence treatment responses. The present investigation delves into the commonly DEGs in HNC and lung cancer, with a particular focus on their significance in lung metastasis. Through a comprehensive suite of analyses, including protein-protein interaction (PPI) networks, hub gene identification, gene ontology enrichment analysis, molecular pathway analysis, survival plot analysis, and comparative mRNA expression level analysis, the study aims to unravel the intricate molecular interplay that underpins the metastatic spread of HNC to the lungs. Insights from such comparisons can drive personalized therapy development, potentially improving survival rates for patients with lung metastasis from HNC.

## Materials and methods

HNSCC samples are analyzed in the Department of Oral and Maxillofacial Surgery, New York University College of Dentistry. Lung squamous cell carcinoma (LSCC) samples were analyzed in the Department of Internal Medicine, University of California, Davis.

Data collection

The raw data for the current study was retrieved from the Gene Expression Omnibus (GEO) database. Constraint keyword and selection criteria have been applied to download the raw data for expression profile array data from a tissue or clinical sample. The accession ID GSE6631 from GEO was used to retrieve expression data for HNSCC and GSE3268 for LSCC. For HNSCC, paired tumor and normal samples from 22 patients were evaluated to identify DEGs. Each pair of samples corresponds to a single patient, providing a direct comparison between tumorous and normal gene expression profiles. In the case of LSCC, the dataset comprises samples from five patients, with each patient contributing two arrays: one derived from cancer cells and the other from normal cells. The DEGs of HNSCC and LSCC were examined for validation using the GEPIA2 online tool (http://gepia2.cancer-pku.cn/#index). Subsequently, the overlapping DEGs of the HNSCC and LSCC were chosen based on the common results from the GEPIA and GEO datasets. This could lessen the effects of the heterogeneity of the various datasets.

Data preprocessing

The Series Matrix Files for GSE6631 and GSE3268 were obtained from the GEO database to enable comprehensive analysis. Before analysis, probe data within each dataset were translated into standard gene symbols, aligning gene identifiers to a universally recognized nomenclature. To ensure uniformity and mitigate potential technical biases, both datasets underwent normalization. This was accomplished using the robust multi-array average method within the R software environment (version 2.6.0), which standardizes gene expression data, ensuring a consistent scale and distribution across the datasets.

Identification of DEGs

In our study, we utilized GEO2R for the analysis of DEGs in HNSCC and LSCC. This tool generated a volcano plot illustrating the fold change in gene expression on the x-axis and the statistical significance (p-value) on the y-axis. To identify DEGs, we set a stringent criterion, requiring a p-value cutoff of <0.05 and an absolute log fold change >1 [11]. To complement this analysis, we downloaded gene expression profiles for HNSCC and LSCC from GEPIA2, applying the same criteria for DEG identification. Additionally, we utilized FunRich v3.1.3 to visualize the overlap and disparities in DEGs across these datasets. The Venn diagram generated by FunRich provides a clear representation of common molecular targets or pathways shared among the datasets.

PPI and hub gene identification

PPI analysis using STRING involves inputting a list of proteins into the database to visualize a network of predicted interactions, which includes both physical and functional associations. A combined score >0.08 was deemed significant for the interactions, denoting the reliability of the protein associations identified. The resulting DEGs were employed to construct and visualize the PPI network using Cytoscape software (version 3.5.1; http://www.cytoscape.org). In this constructed PPI network, the connections between proteins were represented by edges, with widths reflecting the strength of these protein interactions based on the combined score. Hub genes within this network were identified using the Cytohubba plugin in the Cytoscape software. The study identified hub genes, characterized as nodes with a degree >10, indicating their significance within the network [12]. This combined approach provides a comprehensive method for exploring the intricate web of protein interactions and identifying key players in cellular functions.

mRNA expression and survival analysis of hub genes

In silico tools such as UALCAN, GEPIA, and KM plotter were utilized to assess the survival rates and gene expression interactions in patients with HNC and lung cancer. Kaplan-Meier analysis, complemented by log-rank testing, facilitated the survival analysis. A statistically significant association was observed between gene expression levels and patient survival, adhering to a significance threshold of P < 0.05. Data from patients with HNC and lung cancer, sourced from The Cancer Genome Atlas, were employed for expression validation. The data, represented as transcripts per million (TPM) values, allowed for the formation of two distinct groups. These groups were visualized using the GEPIA database, with patients having TPM values below the top quartile categorized into the low/medium expression group and those with TPM values above the upper quartile classified into the high expression group.

Gene ontology and pathway enrichment analysis

Utilizing the Database for Annotation, Visualization, and Integrated Discovery (DAVID, https://david.ncifcrf.gov/tools.jsp), the GO and the KEGG pathway enrichment analysis of DEGs were examined. A p-value of less than 0.05 was used as the cutoff point. Additionally, the study utilized KEGG pathway analysis to identify pathways that were significantly enriched in association with the DEGs. Pathway crosstalk analysis was performed using defined criteria: a Benjamini-Hochberg adjusted (p)-value of less than 0.05 and a Jaccard coefficient combined with an overlap coefficient both set at 50% exceeding (0.5), which was deemed statistically significant. This detailed examination of the DEGs within specific pathways reveals their potential involvement in essential biological processes and regulatory networks.

## Results

Identification of DEGs in HNSCC and LSCC

**Figure 1:**
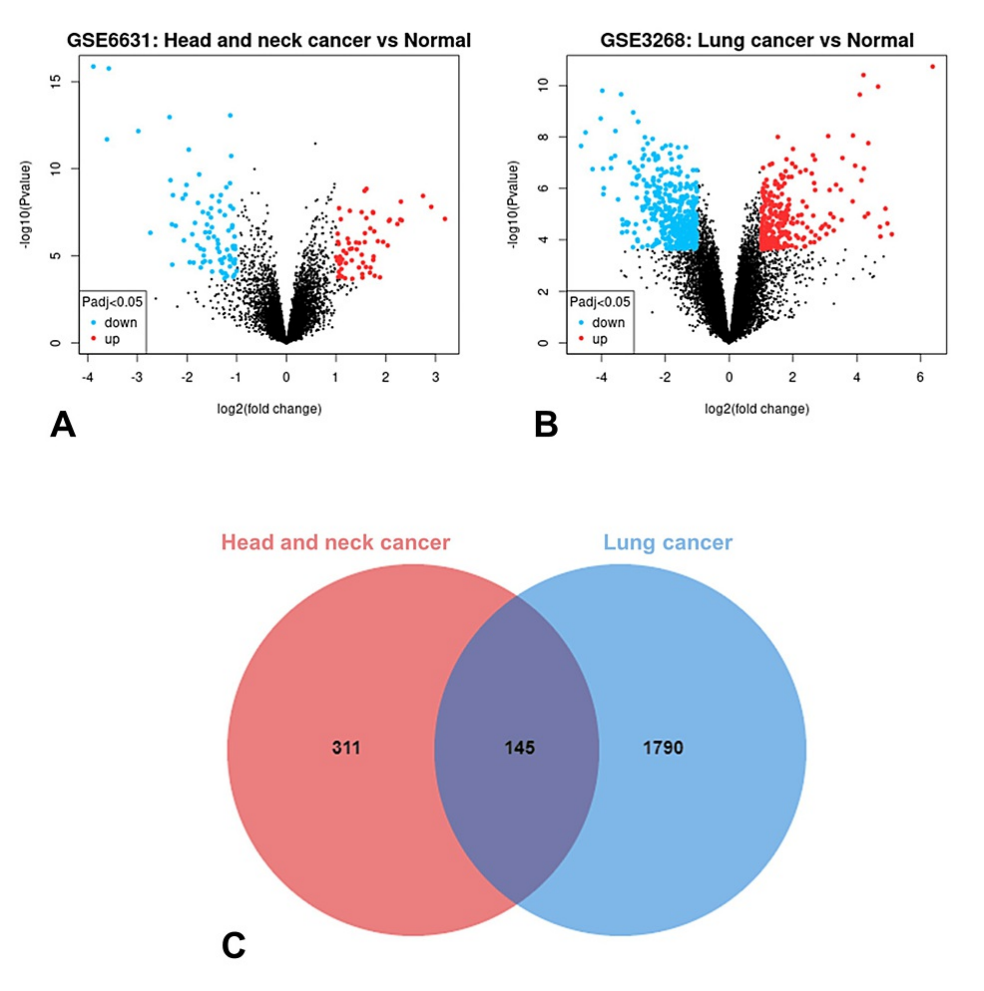
Identification of DEGs Volcano plots depict the DEGs in (A) GSE6631 and (B) GSE3268. (C) The Venn diagram depicts the discovery of 145 overlapping DEGs. DEGs, differentially expressed genes

From the HNSCC cancer dataset GSE6631, 150 DEGs were identified, and from the LSCC cancer dataset GSE3268, 726 DEGs were detected (Figure 1A, 1B). The identification was performed through GEO2R analysis utilizing the Limma package, with stringent selection criteria of an adjusted p-value of <0.05 and a log fold change of >1. Subsequently, this process facilitated the generation of volcano plots for each respective dataset. Similarly, the analysis of the HNSCC dataset from GEPIA identified 405 DEGs. In comparison, the LSCC dataset revealed 1692 DEGs, meeting the threshold of an absolute Log2 Fold Change (|Log2FC|) greater than 1 and a q-value under 0.05. Further examination led to the discovery of 145 overlapping DEGs that could have either promoting or inhibitory effects in both HNSCC and LSCC, as established through the collective data from GEO and the GEPIA database (Figure 1C).

PPI network construction and hub gene identification

**Figure 2:**
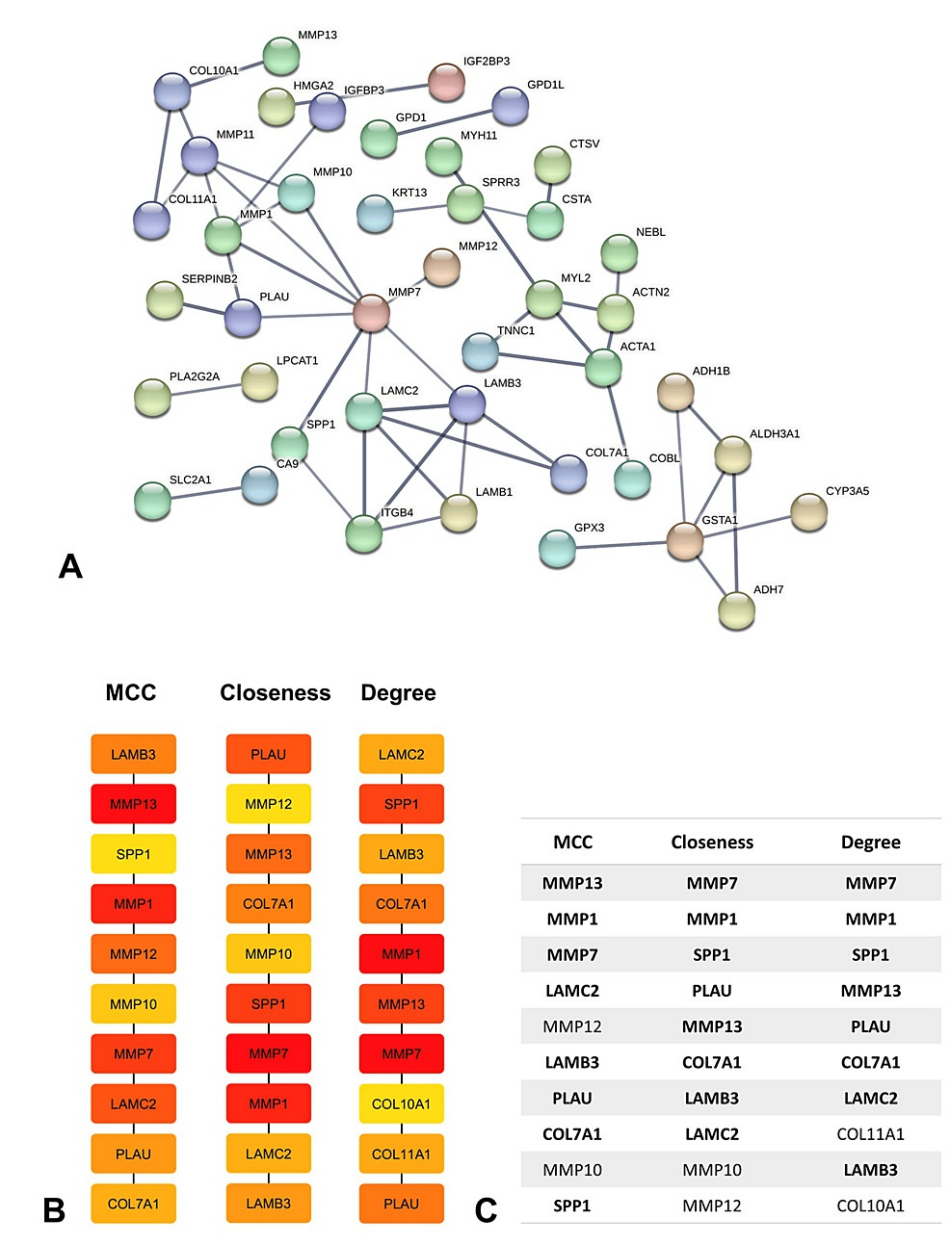
PPI network (A) Interaction network of 145 commonly overlapped genes. (B) Cytoscape cytohubba plugin identified the top 10 hub genes by MCC, degree, and closeness. (C) Cytohubba methods rank hub genes. PPI, protein-protein interaction

The PPI network was established for proteins produced by 145 overlapping DEGs through STRING analysis (Figure 2A). It was observed that 42 out of these 145 DEGs were interconnected, which was further illustrated through Cytoscape visualization. Additionally, eight hub genes (MMP13, MMP1, MMP7, LAMC2, LAMB3, PLAU, COL7A1, and SPP1) were identified using methods such as MCC, closeness, and degree centrality (Figure 2B, 2C). Remarkably, all these hub genes showed upregulation in the overlapping DEGs, suggesting they may have significant roles in the development of both HNSCC and LSCC.

Gene ontology and KEGG pathway analysis of DEGs

**Figure 3:**
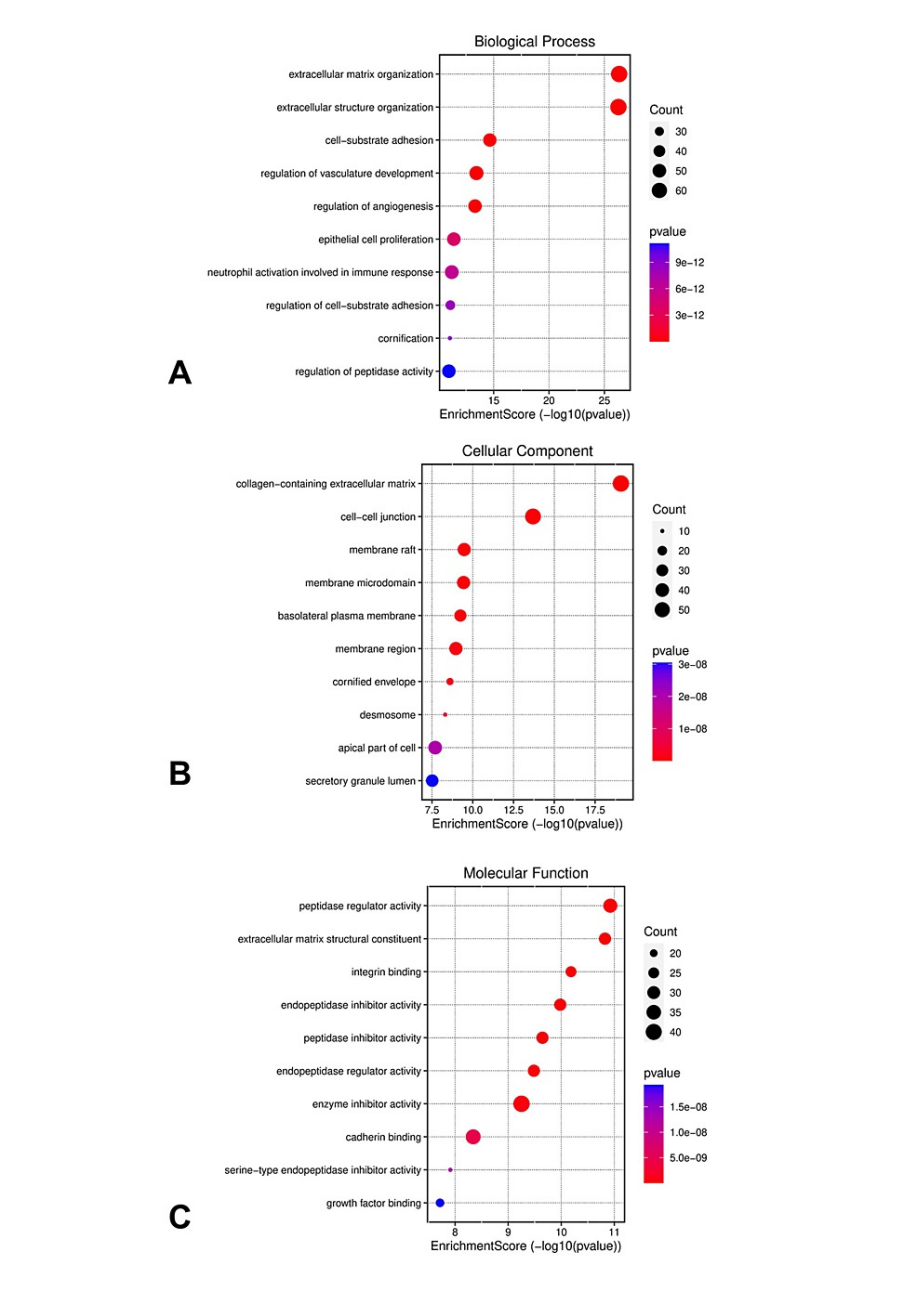
Gene ontology analysis of DEGs in both HNC and lung cancer (A) Biological process. (B) Cellular component. (C) Molecular function. DEGs, differentially expressed genes; HNC, head and neck cancer

**Figure 4:**
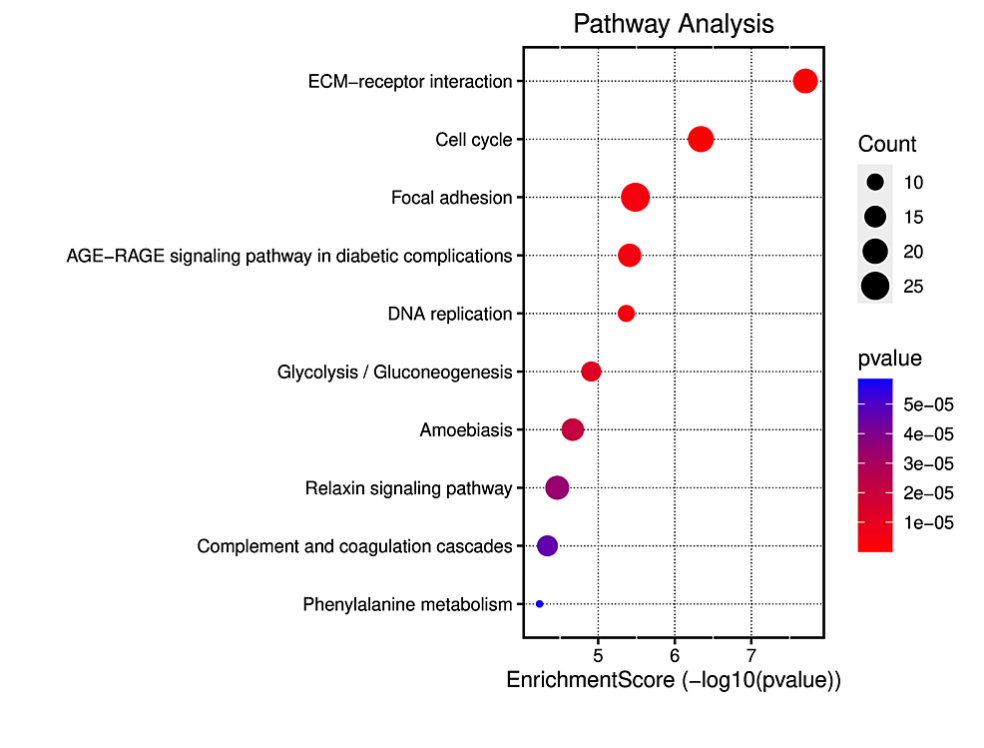
Pathway analysis of DEGs DEGs, differentially expressed genes; ECM, extracellular matrix

**Figure 5:**
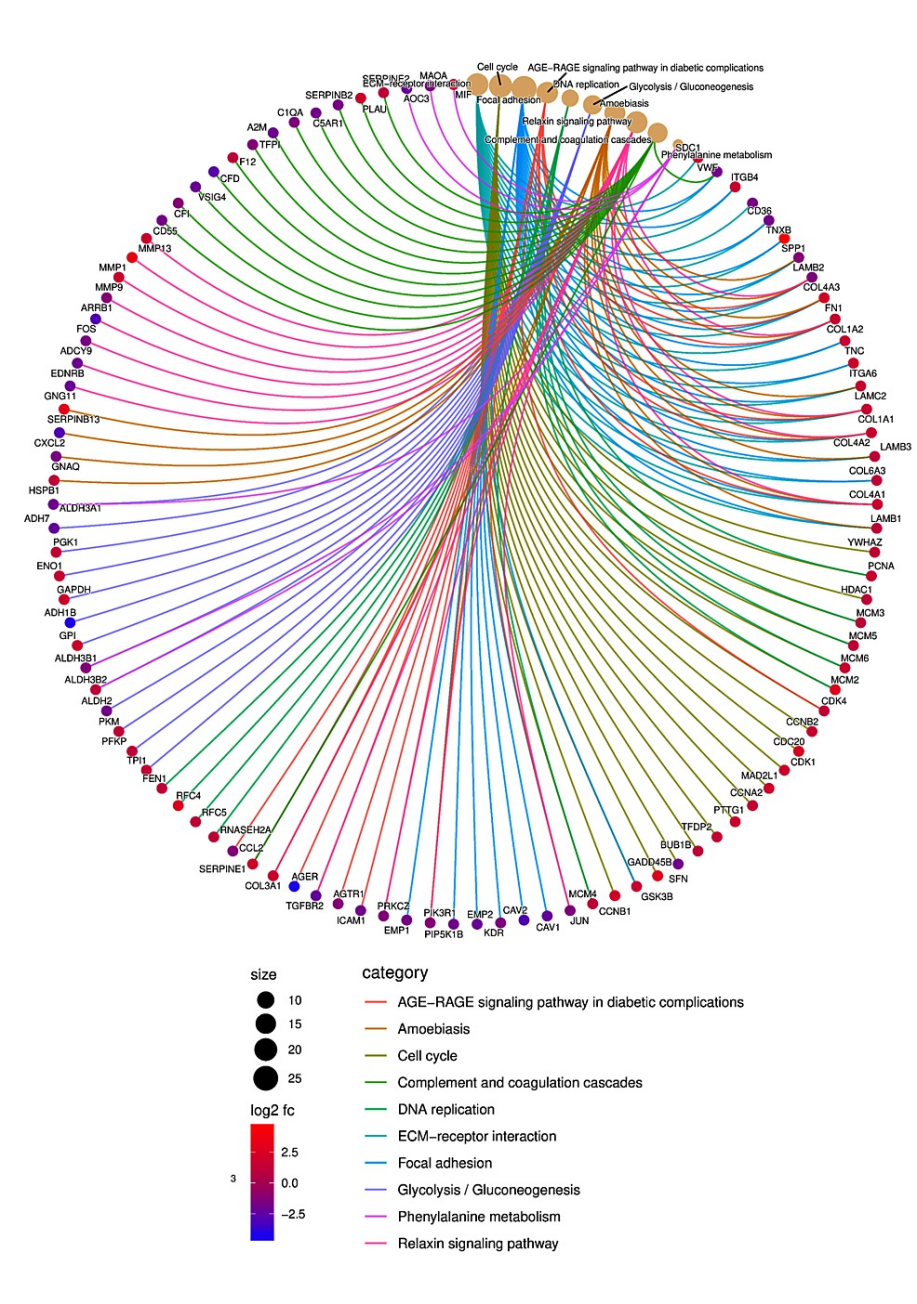
Cnet plot of DEGs DEGs, differentially expressed genes; ECM, extracellular matrix

The gene ontology analysis for HNSCC and LSCC highlights several biological processes, cellular components, and molecular functions that are intricately linked to the prognosis for lung metastasis in HNC patients. The enrichment of genes in cellular matrix organization and cell-substrate adhesion suggests a pivotal role in the detachment and invasion of cancer cells, facilitating their dissemination from the primary tumor site to the lungs. Epithelial cell proliferation indicates rapid tumor growth, a hallmark of aggressive cancers with a higher metastatic potential. Neutrophil activation within the immune response may contribute to a microenvironment that either supports or inhibits metastatic spread, depending on the balance of pro- and anti-tumor factors (Figure 3A). From a cellular component perspective, the collagen-containing extracellular matrix (ECM) and cell-cell junctions are essential for maintaining tissue architecture; their disruption could enable metastatic escape. Membrane rafts and microdomains, along with basolateral plasma membrane and desmosome structures, are implicated in signal transduction and cellular adhesion, both critical for the metastatic process (Figure 3B). Molecular functions such as integrin binding, cadherin binding, and growth factor binding reflect the complex interactions between cells and their microenvironment, influencing cell migration and survival during metastasis. Peptidase regulator and inhibitor activities, by modulating proteolytic processes, play a role in ECM remodeling, a necessary step for metastatic progression (Figure 3C). The pathway enrichment analysis of DEGs in HNSCC and LSCC underscores the complexity of lung metastasis in HNC. The ECM-receptor interaction and focal adhesion pathways are integral to cell migration and adhesion, facilitating the detachment and spread of cancer cells. The cell cycle and DNA replication pathways indicate increased cellular proliferation, a trait of metastatic cells. Glycolysis reflects the metabolic reprogramming of cancer cells, supporting their survival and proliferation. The relaxin signaling pathway modulates the tumor microenvironment, promoting metastasis. Complement and coagulation cascades could enhance the survival of circulating tumor cells, while phenylalanine metabolism contributes to the biosynthesis of compounds aiding tumor growth and metastasis (Figure 4 and Figure 5). These pathways collectively reveal potential mechanisms and therapeutic targets for preventing lung metastasis in HNC patients.

Verification and survival analysis of hub genes in HNSCC and LSCC

**Figure 6:**
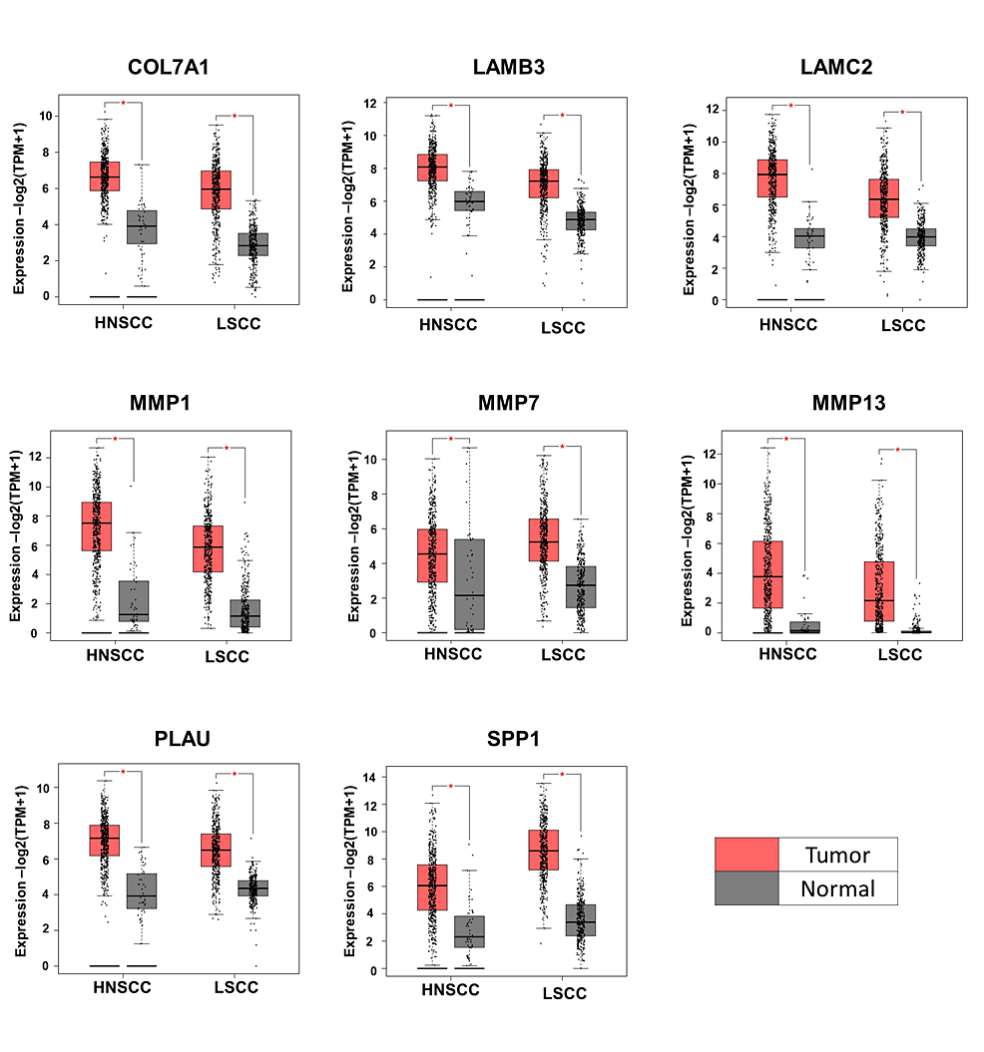
The mRNA expression of hub genes in HNC and lung cancer The expression levels of these hub genes were compared between cancer patient samples (in red) and normal samples (in gray). HNC, head and neck cancer; HNSCC, head and neck squamous cell carcinoma; LSCC, lung squamous cell carcinoma

**Figure 7:**
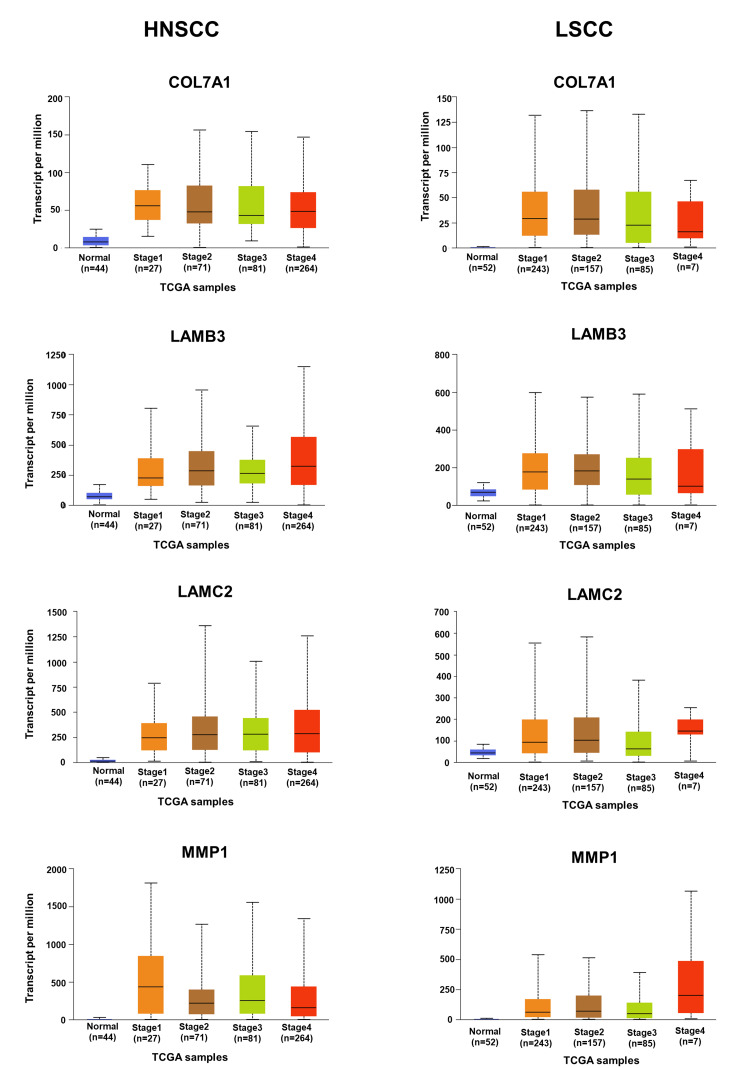
Stages of HNSCC and LSCC relevant to expression of hub genes (COL7A1, LAMB3, LAMC2, and MMP1) HNSCC, head and neck squamous cell carcinoma; LSCC, lung squamous cell carcinoma

**Figure 8:**
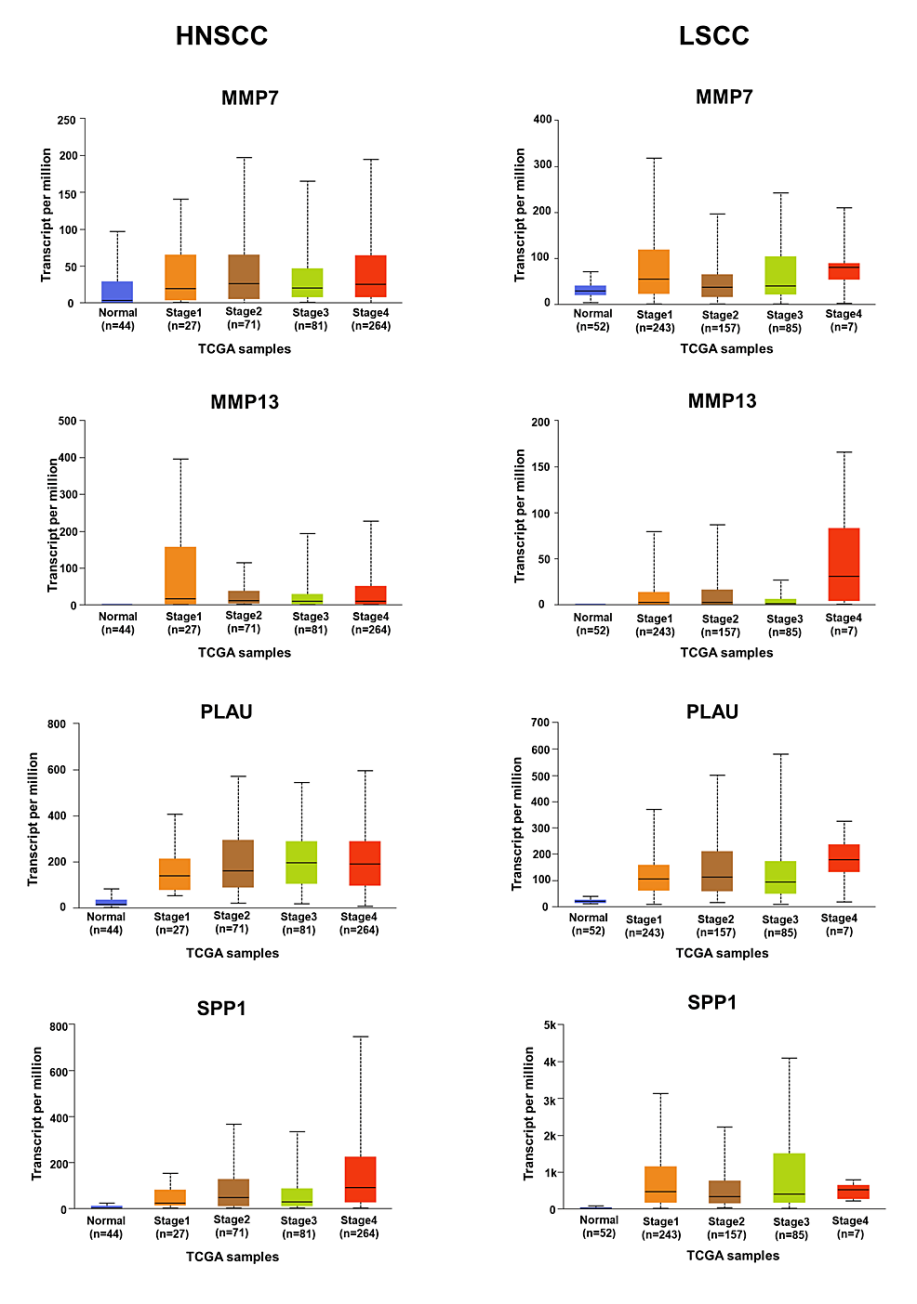
Stages of HNSCC and LSCC relevant to the expression of hub genes (MMP7, MMP13, PLAU, and SPP1) HNSCC, head and neck squamous cell carcinoma; LSCC, lung squamous cell carcinoma

**Figure 9:**
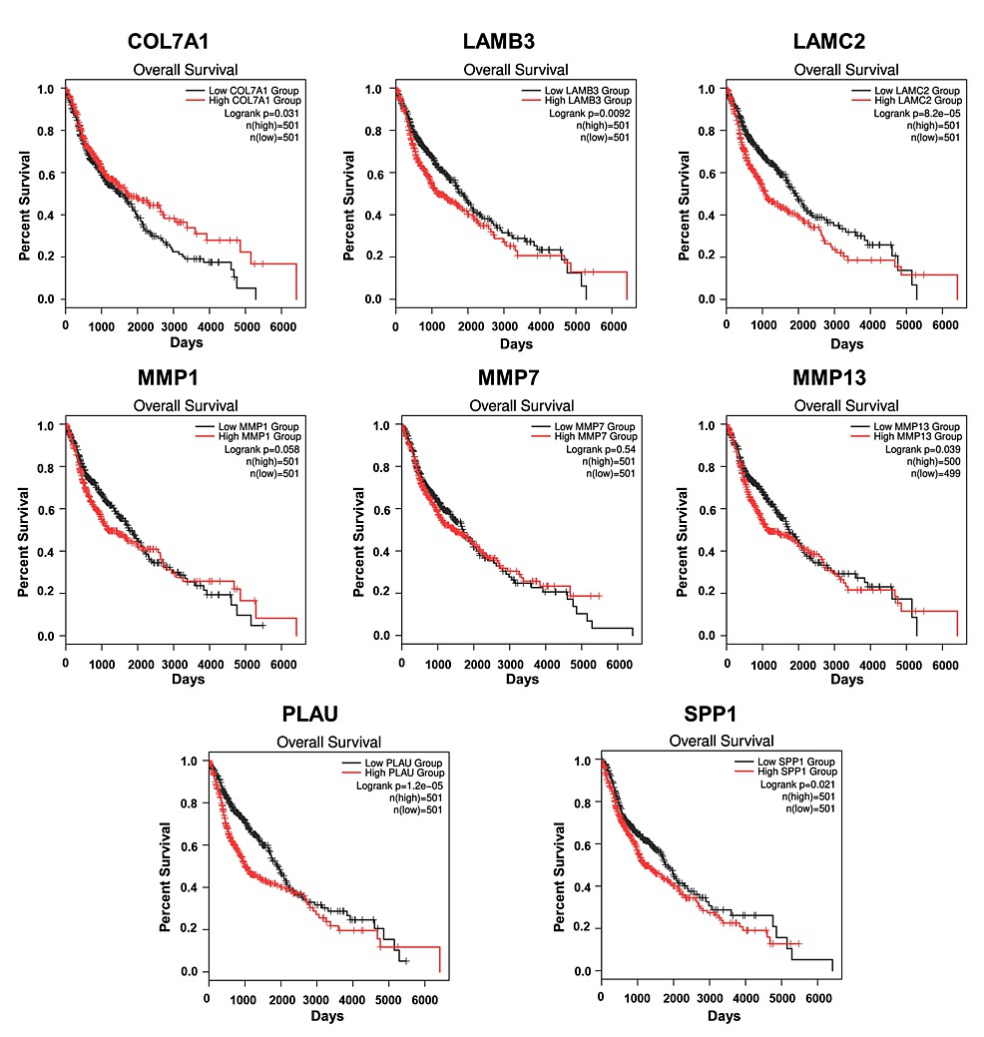
Overall survival analysis of the hub genes

A comparative mRNA expression analysis of hub genes was performed for HNSCC and LSCC using the GEPIA platform. The study revealed a notable upregulation of all eight hub genes in both cancer types, which is illustrated in Figure 6. This upregulation is particularly significant as it may contribute to the prognosis of lung metastasis in HNC patients. The consistent overexpression of these genes in both HNSCC and LSCC could provide insights into the molecular pathways that facilitate the spread of cancer cells to the lungs, which is the most frequent site of distant metastasis from HNC. The correlation between the expression levels of key hub genes and the stages of patients’ cancer was investigated using the UALCAN platform (Figure 7 and Figure 8). The observed upregulation of hub genes in both grade 2 and grade 3 HNSCC, as well as in stage 2 and stage 3 LSCC, suggests a correlation with the progression of these cancers. This gene expression pattern indicates an enhanced capacity for tumor invasion and metastasis, particularly to the lungs, as the disease advances. The consistent increase across different grades and stages implies that these genes could be integral to the underlying mechanisms driving the metastatic process, potentially serving as biomarkers for aggressive disease and as targets for therapeutic intervention aimed at mitigating lung metastasis in HNC patients. The overall survival analysis rate of HNSCC and LSCC patients in TCGA was performed based on hub genes using the UALCAN (Figure 9). In the context of HNSCC and LSCC, the survival analysis reveals that high gene expression of COL7A1, LAMC2, LAMB3, MMP13, PLAU, and SPP1 significantly correlates with patient outcomes, as evidenced by p-values less than 0.05. The extremely low p-values for LAMC2 (8.2e-05) and PLAU (1.2e-05) suggest a particularly strong association with survival, highlighting their potential as critical biomarkers for aggressive disease progression. LAMB3, COL7A1, MMP13, and SPP1 also demonstrate significant associations, with p-values of 0.0092, 0.031, 0.039, and 0.021, respectively, indicating their relevance in patient prognosis. In contrast, the expression of MMP1, exhibiting a p-value of 0.058, approaches statistical significance, suggesting a possible yet inconclusive influence on patient survival outcomes. This indicates that while MMP1 may contribute to the prognostic landscape, its impact is not as clearly defined as those genes with p-values firmly below the threshold of 0.05. MMP7, however, with a p-value of 0.54, does not exhibit a statistically significant correlation, suggesting its expression may not be a reliable indicator of survival in these cancers. Collectively, these results underscore the importance of these genes in the pathophysiology of HNSCC and LSCC, with LAMC2 and PLAU standing out as particularly promising targets for further research and potential therapeutic intervention. By customizing treatments based on each patient’s unique genetic profile, personalized medicine techniques may be made possible by the results of this study.

## Discussion

Despite the considerable progress in elucidating the pathogenesis of HNC recently, the clinical outlook for individuals afflicted with pulmonary metastases continues to be poor. The lungs are the most common site of distant metastasis from HNC, with the incidence of clinical metastases reported to be between 4% and 25%. A study found that the rate of distant metastasis at presentation is 4.73%, with the lung being the most common site [13]. Moreover, nasopharyngeal malignancy cases showed the highest frequency of distant metastasis (29.03%), whereas oral cavity patients had the lowest (2.75%). The most common site of distant metastasis was the lung (64%), followed by bone (18%) and liver (11%) [13]. Identifying particular molecular markers that indicate the growth and spread of lung cancer is crucial for diagnosing and treating HNC.

In this study, analysis of the HNSCC and LSCC datasets revealed 150 and 726 DEGs, respectively. Further investigation identified 145 overlapping DEGs, with 42 forming a PPI network. Eight hub genes (MMP1, MMP7, MMP13, LAMC2, LAMB3, PLAU, COL7A1, and SPP1) were upregulated in both HNSCC and LSCC, suggesting a significant role in cancer progression. Matrix metalloproteinases (MMPs), particularly MMP13, MMP1, and MMP7, are known to play a significant role in cancer progression. These enzymes are involved in degrading the ECM, which not only facilitates tumor invasion and metastasis but also promotes angiogenesis and evasion of the immune system. Studies have shown that these MMPs are often upregulated in most cancer types, contributing to the cancer cell’s ability to invade and migrate to other parts of the body [14]. MMP13 degrades collagen and other matrix components, which is crucial for the invasive potential of cancer cells. Similarly, MMP1 and MMP7 contribute to the breakdown of the ECM, allowing cancer cells to penetrate tissue barriers and spread. The upregulation of these MMPs has been observed in HNSCC, indicating their importance as potential therapeutic targets and prognostic biomarkers [15]. The PLAU gene, encoding the urokinase plasminogen activator (uPA), plays a significant role in lung metastasis in HNC patients. While its primary function is to break down blood clots, uPA also facilitates cancer cell invasion and migration [16]. The interaction between uPA and its receptor, uPAR, triggers a cascade of proteolytic activities that not only enable tumor cells to invade the ECM but also assist in their migration through the bloodstream to distant organs [17]. The overexpression of uPA has been associated with increased tumor aggressiveness and a higher propensity for lung metastasis.

Moreover, research has shown that uPA interacts with other proteins, such as MMP1, to promote the proliferation, invasion, and metastasis of HNC cells [18]. This suggests that the PLAU gene’s role in cancer progression is multifaceted and involves complex interactions with various molecular pathways. The SPP1 gene, which encodes osteopontin, is significantly implicated in the process of lung metastasis from HNC [19]. Studies have shown that osteopontin is often overexpressed in HNC and is associated with increased tumor aggressiveness and a higher likelihood of metastasis to distant organs, including the lungs [20]. Its role in promoting tumor growth and metastasis is linked to its ability to interact with cell surface receptors, such as integrins and CD44, which initiate signaling pathways that lead to the proliferation and survival of cancer cells, as well as their movement and invasion. Inhibiting the function of osteopontin could potentially reduce the metastatic spread of HNC to the lungs, offering a promising avenue for improving patient outcomes [21].

The enrichment of pathways related to cell migration and adhesion (ECM-receptor interaction and focal adhesion) underscores the critical role of cellular detachment and dissemination in metastasis. A recent study demonstrated that high expression of integrin β1, a key ECM receptor, is associated with a poor prognosis and an increased risk of lung metastasis in HNSCC patients [22]. Another study by Chuang et al. found that focal adhesion kinase interacts with other signaling pathways, such as PI3K/AKT and MAPK, to promote lung metastasis in HNSCC [23]. The involvement of cell cycle and DNA replication pathways implicates accelerated proliferation, a hallmark of metastatic tumors. Other cell cycle regulatory proteins, such as p21 and MDM2, have altered expression levels that are associated with lung metastasis development in HNSCC patients [24]. The observed enrichment of glycolysis suggests a reprogrammed metabolic landscape favoring aerobic glycolysis, even in the presence of oxygen. This metabolic switch fuels tumorigenesis by providing biosynthetic precursors and ATP for rapid proliferation and survival [25].

Limitation

One limitation of this study is the reliance on retrospective data from public databases, which may not fully represent the heterogeneity of the patient population. Additionally, the bioinformatics tools used, while robust, cannot substitute for experimental validation in a clinical setting. The identification of DEGs and hub genes, although suggestive of their roles in HNSCC and LSCC progression, requires further investigation to establish causality. Moreover, the PPI network analysis is limited by the current understanding of protein interactions, which is continually evolving. Consequently, the findings should be interpreted with caution and considered as a foundation for future research.

## Conclusions

This study investigated the underlying molecular mechanisms of lung metastasis in HNSCC and LSCC. By analyzing gene expression patterns, we identified 145 DEGs common to both cancers, potentially playing crucial roles in the metastatic spread. Functional analysis revealed enrichment in processes like cell adhesion, matrix remodeling, and epithelial proliferation, all linked to metastasis. Further network analysis pinpointed eight hub genes, exhibiting upregulation in both HNSCC and LSCC. Notably, increased expression of these hub genes correlated with advanced cancer stages and decreased patient survival, particularly LAMC2 and PLAU, suggesting their potential as prognostic biomarkers. Overall, this work provides insight into the complex molecular mechanisms of lung metastasis in head and neck malignancies, identifying several genes and pathways that could be potential targets for future diagnosis, therapy, and personalized medicine strategies.
